# Shifting focus from resistance to disease tolerance: A review on hybrid house mice

**DOI:** 10.1002/ece3.8889

**Published:** 2022-05-07

**Authors:** Alice Balard, Emanuel Heitlinger

**Affiliations:** ^1^ Department of Molecular Parasitology Institute for Biology Humboldt University Berlin (HU) Berlin Germany; ^2^ Research Group Ecology and Evolution of Molecular Parasite‐Host Interactions Leibniz‐Institut for Zoo and Wildlife Research (IZW) im Forschungsverbund Berlin e.V. Berlin Germany

**Keywords:** *Eimeria*, hybridization, *Mus musculus*, pinworms, resistance, tolerance

## Abstract

Parasites have been proposed to modulate the fitness of hybridizing hosts in part based on observations in the European house mouse hybrid zone (HMHZ), a tension zone in which hybrids show reduced fitness. We here review evidence (1) for parasite load differences in hybrid versus parental mice and (2) for health and fitness effects of parasites promoting or preventing introgression and hybridization. The question of relative resistance or susceptibility of hybrids to parasites in the HMHZ has long been controversial. Recent field studies found hybrids to be more resistant than mice from parental subspecies against infections with pinworms and protozoans (*Eimeria* spp.). We argue that the field studies underlying the contradictory impression of hybrid susceptibility have limitations in sample size, statistical analysis and scope, focusing only on macroparasites. We suggest that weighted evidence from field studies indicate hybrid resistance. Health is a fitness component through which resistance can modulate overall fitness. Resistance, however, should not be extrapolated directly to a fitness effect, as the relationship between resistance and health can be modulated by tolerance. In our own recent work, we found that the relationship between health and resistance (tolerance) differs between infections with the related species *E*.* falciformis* and *E*.* ferrisi*. Health and tolerance need to be assessed directly and the choice of parasite has made this difficult in previous experimental studies of house mice. We discuss how experimental *Eimeria* spp. infections in hybrid house mice can address resistance, health and tolerance in conjunction.

## Graphical abstract

Our review shows evidence for hybrid vigor against in the house mouse hybrid zone. This vigor manifests in elevated resistance. We discuss that resistance is not necessarily a linked with fitness and argue that this research should switch its focus to tolerance. We describe house mice and protozoan parasites of the genus *Eimeria* as a host–parasite system in which this is possible.

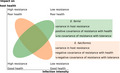

## 1 INTRODUCTION

There has been a long‐standing debate about whether house mouse hybrids are more susceptible or resistant to parasites than their parents, and whether parasites can modulate hybrid fitness. In the light of contradictory results arising from the field studies, we argue that an in depth review and re‐evaluation of research procedures is timely and even necessary. Additionally, the limited number of available experimental infection studies on hybrid mice use inconsistent approaches and provide conflicting results. This disjunct literature in the field might hamper progress. The first aim of our review was to present and discuss the work surrounding resistance/susceptibility of house mouse hybrids in response to parasites, including statistical analyses and conceptual approaches.

To determine whether parasites modulate the fitness of hybrids, studying resistance alone is not sufficient: one needs to investigate the host's ability to resist AND tolerate parasitism, as observing a low or high parasite load alone does not mean that a host is more or less healthy (or fertile). Resistance, tolerance, and trade‐offs between the two have been reviewed from multiple perspectives with a broad focus on concepts. Using research on these concepts, the second aim of the present review was to discuss the importance of including tolerance to studies aiming at examining a potential impact of parasites on host fitness.

With this review we aim to inspire others and help to revive house mice as an experimental system for research on the effects of hybridization on parasitemia. We also hope a review driven by an example of one major host–parasite system can be helpful for the reader interested in the general concepts, and provides a specific example of how resistance and tolerance could be studied more fruitfully in the context of hybridization.

## 2 THE IMPORTANCE OF HYBRIDIZATION AND THE EUROPEAN HOUSE MOUSE HYBRID ZONE

Hybridization is the recombination between previously isolated populations, for example, between species (Barton & Hewitt, 1985; Mallet, 2005). Traditionally, hybrids were thought of as a rarity, but it seems now that a large proportion of plants (10%) and animals (25%) can produce hybrids in nature (Mallet, 2005). Not only does the study of hybrids allow us a better understanding of the mechanisms of speciation, but it also has relevance for the present and future of biodiversity: hybridization with introduced species can threaten autochthonous endangered animals, making studies of hybridization relevant for conservation biology (Simberloff, 1996). Hybridization, however, can also lead to the emergence of new taxa and Stronen and Paquet (2013) argue that the ecological role of hybrids could justify their protection by conservation policies, in the same way that taxa which are considered as species according to an ecological species concept. Moreover, hybrid zones represent melting pots of genotypes that allow for exploration of the impact of genetic diversity and specific genetic configurations on different physiological systems (e.g., reproduction and immunity).

**FIGURE 1:**
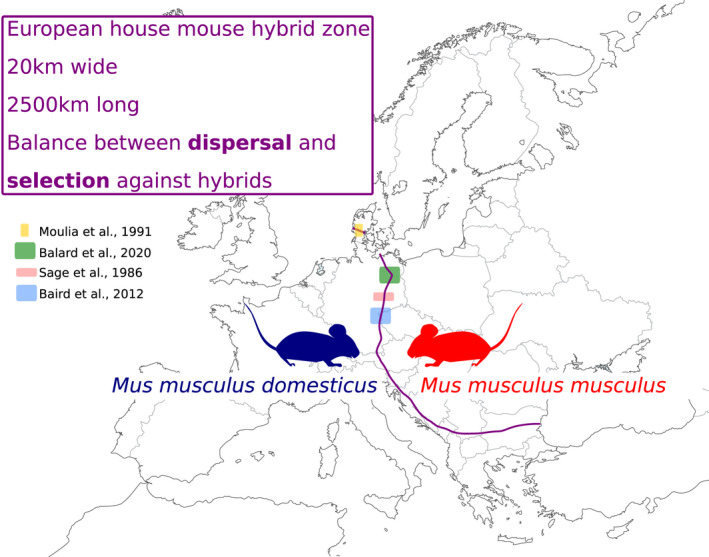
Approximate course of the European house mouse hybrid zone (purple line) between *Mus musculus domesticus* (blue) and *Mus musculus musculus* (red) areas (adapted from Baird et al. (2012)). The colored squares show the location of transects investigated for hybrid parasite load (Baird et al., 2012; Balard, Jarquín‐Díaz, Jost, Martincová, et al., 2020; Moulia et al., 1991; Sage et al., 1986, as indicated in the legend see also Table 1)

The house mouse (*Mus musculus*) is the most widely used animal model in biomedicine. The vast majority of inbred lines used in contemporary studies, however, are not “natural” animals: they originate from mice bred as pets in the late 19th and beginning of 20th century. They are mixtures of four different subspecies (Davisson & Linder, 2004; Yang et al., 2011). The common ancestor to all *Mus musculus* subspecies originates from the Indo‐Pakistani region. Several subspecies (or species, depending on one's views and usage of species concepts; Boursot et al. (1993)) emerged after range expansions from this cradle, having diverged (mostly) in allopatry for about half a million years (Duvaux et al., 2011; Geraldes et al., 2008, 2011). At least five subspecies have been described based on phylogenetic analysis of morphological (Boursot et al., 1993) and genetic data (from single marker genes, e.g., CytB (Suzuki et al., 2013) to whole genomes (Yang et al., 2011): *M*.* m*.* musculus*, *M*.* m*.* domesticus*, *M*. *m*. *castaneus*, *M*. *m*. *molossinus*, and *M*. *m*. *gentilulus*). As commensal mice were following human migrations (Boursot et al., 1993), there is a wide range of evidence that these subspecies are not in complete reproductive isolation, and that gene flow occurs between them in zones of secondary contact (Auffray & Britton‐Davidian, 2012). In Europe, *M*.* m*.* domesticus* (hereafter Mmd) and *M*.* m*.* musculus* (hereafter Mmm) entered into secondary contact around the Bronze Age after having taken different colonization routes, respectively south and north of the Black Sea (Bonhomme & Searle, 2012), this secondary contact led to the formation of a hybrid zone that is about 20 km wide and more than 2500 km long, running from Denmark to the Black Sea: the European house mouse hybrid zone (HMHZ) (Baird & Macholán, 2012; Boursot et al., 1993) (Figure 1). Despite hybridization, Mmd and Mmm subspecies show stable differences in several traits including pelage color, tail/body length ratio (shorter for Mmm than for Mmd) (Boursot et al., 1993), boldness and activity (Frynta et al., 2018), and male aggressiveness (Ďureje et al., 2010).

Through the HMHZ, gene flow between both subspecies is not completely interrupted, and introgression from one side to the other occurs (Macholán et al., 2007, 2011, 2019; Payseur et al., 2004; Raufaste et al., 2005). Hybrids between Mmd and Mmm are highly recombinant, presenting a range of genotypes, and no F1 or early‐generation hybrids are found (Macholán et al., 2007). Studies performed on geographically independent transects of the HMHZ give strong support to the tension zone model in this system: the immigration of less hybridized mice to the center of the zone, increasing the hybrid population size, is balanced by endogenous selection against hybrids (Barton & Hewitt, 1985). This negative selection on hybrids seems to be linked with reduced fertility and especially disruption of spermatogenesis has been shown (Albrechtová et al., 2012; Martincová et al., 2019; Turner & Harr, 2014; Turner et al., 2012). Reduced fertility has very immediate fitness consequences showing how genetic incompatibilities reduce fitness without the influence of extrinsic factors.

## 3 A LONG‐LASTING CONTROVERSY: PARASITES AS A SELECTIVE FACTOR FOR HOSTS?

**FIGURE 2:**
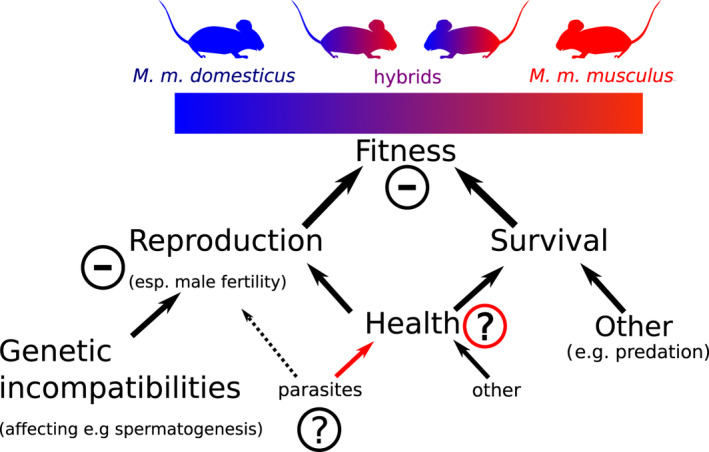
Hybrid fitness is reduced in the house mouse hybrid zone (HMHZ). Reproduction is negatively affected in hybrids, for example, via disruption of spermatogenesis (Martincová et al., 2019). This reduction in reproductive fitness is a direct consequence of genetic incompatibilities without the involvement of extrinsic factors. The fitness effect of health reductions by parasites can be mediated via (additional) effects on reproduction or by effects on survival. A direct effect of parasites on reproduction is not plausible for the parasites of the HMHZ but possible for parasites of other hosts. The overall fitness of hybrid mice combined from all these components is reduced compared to mice from parental subspecies. Minus symbols are depicted for well‐established differences between hybrids and parental subspecies. Question marks represent research areas with controversial results (black circle) or the need for further research (red circle): Whether parasite loads are higher or lower in hybrids of the HMHZ is a long‐lasting debate and the first topic of this review. How parasites (and potential load differences) affect health (read arrow) is developed in later paragraphs

Parasites are ubiquitous in natural systems and impose fitness consequences on their hosts, subsequently resulting in hosts exhibiting selection to better defend themselves (Schurer et al., 2016). Their close interaction with their hosts over several generations makes them a source for natural selection pressure. The host immune system as a whole is shaped by pathogens as a selective force (Schmid‐Hempel, 2011). For this reason parasitic infections have been hypothesized to be a potential modulator of the fitness of hybridizing hosts (Figure 2).

The HMHZ is the first animal hybrid zone studied for differences in parasite loads and findings seemed to indicate elevated worm load in hybrids (Sage et al., 1986). These results were interpreted as a consequence of hybrid incompatibilities: after having evolved separately within each subspecies, coadapted gene complexes of the immune system would have been broken down in hybrids, which would lead to reduced fitness (Moulia et al., 1991, 1993; Sage et al., 1986). A second study with slightly larger sample sizes soon confirmed the findings in an independent transect of the HMHZ (Moulia et al., 1991). These findings and a proposal of an extrinsic effect stabilizing species barriers were in contradiction with most proponents of tension zone models, as population genetics considers breakdown of co‐adapted complexes to primarily have effects within the genome independent of the environment (Barton & Hewitt, 1985).

The idea that increased susceptibility to parasites reduced hybrid fitness was attractively controversial. Further studies, however, did not support the early results: in 2012 a much larger field study showed that hybrids have reduced helminth loads compared to parental subspecies (Baird et al., 2012). The same result (with a focus on parasite intensity instead of abundance, see below) was then corroborated for pinworms in a different transect and additionally for *Eimeria* spp. as an intracellular parasite recently (Balard, Jarquín‐Díaz, Jost, Martincová, et al., 2020). It may prove helpful to review previous studies on hybrid resistance in this system in detail and attempt to evaluate the evidence for at least the basic direction of a hybrid effect on parasite infection. Do we find more resistant or more susceptibility to parasites in hybrid house mice? In Table 1 we summarize the key characteristics of each study explicitly addressing differences between hybrid and parental subspecies parasite load in the HMHZ.

**TABLE 1:** Field studies addressing relative parasite load of hybrids compared to parental subspecies in the HMHZ. In the column “Parasite and its characteristics” we use virulence in the sense of pathogenicity, the expected strength of an effect on host health

References	Parasite and its characteristics	Origin of mice	Number of mice (number of localities)	Hybrid definition	Parasite load measurement and statistical test	Result
Sage et al. (1986)	Digestive helminths (low virulence)	Central Eastern Germany	93 (30)	**Categorical** Hybrid index based on 4 diagnostic markers Hybrid = HI between 12.5% and 87.5% of Mmd introgression	Two abundance categories (wormy/not wormy) Chi square test	Hybrid susceptibility
Moulia et al. (1991)	Digestive helminths (low virulence)	Denmark (NB: mice kept 2 months in the laboratory before sacrifice and parasite count)	120 (12)	**Categorical** Hybrid index based on 10 diagnostic markers Hybrid = HI between 20% and 60% of Mmd introgression	Individual parasite abundance Kruskal‐Wallis test & Noether's post‐hoc test between Mmd, Mmm & hybrid	Hybrid susceptibility
Baird et al. (2012)	Digestive helminths (a range of virulence)	South Eastern Germany & Czech Republic (South of Sage et al.)	689 (107)	**Continuous** Hybrid index based on 1401 diagnostic markers	Individual parasite abundance Maximum likelihood estimation along the hybrid index	Hybrid resistance
Balard, Jarquín‐Díaz, Jost, Martincová, et al. (2020)	Digestive helminths & coccidia (large range of virulence)	North Eastern Germany (North of Sage et al.)	650 (149)	**Continuous** Hybrid index based on 14 diagnostic markers	Individual parasite abundance and intensity Maximum likelihood estimation along the hybrid index	Hybrid resistance

Note

Red: hybrid susceptibility; Green: hybrid resistance.

As a first difference between these studies, we note a seeming change over time, from hybrid susceptibility to hybrid resistance. In particular, the two field studies finding hybrid susceptibility (Moulia et al., 1991; Sage et al., 1986) rely on data collected about 20 years earlier than the two field studies finding hybrid resistance (Baird et al., 2012; Balard, Jarquín‐Díaz, Jost, Martincová, et al., 2020). One could suspect a change of hybrid response to parasites in terms from susceptibility to resistance over time. Indeed, Wolinska et al. (2008) proposed that parasites could represent a dynamic selective force in hybrid zones. Frequency‐dependent selection could then explain oscillations between hybrid resistance and hybrid susceptibility scenarios. According to this model, parasites would adapt alternatively to the most common host taxon, represented either by parents or by hybrids. If parasites decrease host fitness, the relatively more infected host taxon decreases in prevalence. Eventually the other taxon could become the more common one, targeted by parasites, and this cycle could be repeated. As noted by Baird et al. (2012), however, the HMHZ system has no F1 and early generations hybrids: late generation, highly recombinant hybrids represent a highly diverse genetic pool of individuals rather than one homogeneous “hybrid taxon.” Thus, in our opinion, this frequency‐dependent selection dynamic is unlikely to apply to the HMHZ.

As a second difference, geographical discrepancies could explain differences in resistance or susceptibility of hybrids. Indeed, a broad range of geographical locations (Figure 1 and Table 1 column “Origin of mice”) has been studied. This, however, is still hard to conciliate with the fact that the two more recent studies finding hybrid resistance against the same parasites (Baird et al., 2012; Balard, Jarquín‐Díaz, Jost, Martincová, et al., 2020), were performed in regions in both Northern and Southern proximity to the initial study finding hybrid susceptibility (Sage et al., 1986).

**FIGURE 3:**
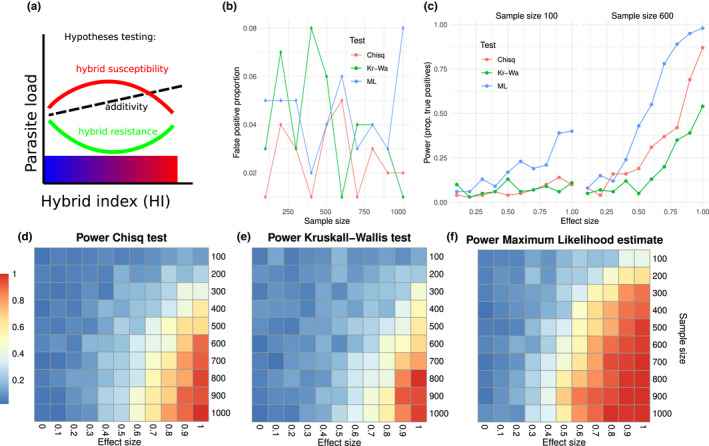
Hybridization expressed and modelled as a continuous index increases statistical power. (a) Conceptual depiction of the analysis developed by Baird et al. (2012) and applied in Balard, Jarquín‐Díaz, Jost, Martincová, et al. (2020). A continuous hybrid index (HI) is used to test whether hybrid mice present higher or lower parasite burdens than expected in case of additivity: if the relationship between parasite load and HI is linear, hybrids have an intermediate parasite load between those of the parental subspecies. A “bent parameter,” representing the strength of the deviation from additivity, is estimated in a maximum likelihood (ML) approach. This parameter can be visualized as the strength of the curvature in one or the other direction (susceptibility or resistance), and represents the effect size of the statistical test. The formula for this polynomial (“bent”) is derived from expected heterozygosity linking HI to the amount of genetic mixing. For b–f we simulated 100 datasets each for sample sizes ranging from 100 to 1000 from a negative binomial distribution with a mean of 100 affected by a “bent parameter” (effect sizes) from 0 (no effect of hybridization on resistance/susceptibility) to 1 (strong effect of hybridization). We used a distribution of mice along the hybrid index sampled from Baird et al. (2012) and Balard, Jarquín‐Díaz, Jost, Martincová, et al. (2020) to represent the usually uneven sample across HI. We then applied the Chi‐square test of Sage et al. (1986), the Kruskall–Wallis test of Moulia et al. (1991) or the ML estimation of Baird et al. (2012), to compare the power of these different tests according to effect and sample sizes. (b) shows the proportion of false positive tests in the absence of an effect of hybridization on resistance/susceptibility (effect size = 0, which corresponds to the first column of the heatmaps d–f). The tests do not differ in statistical specificity as false positive proportions are similar. (c) Shows the power as a function of effect size, for sample size of 100 (as approximately used in the two early studies and when focusing on *Eimeria* intensity) and 600 (as used in recent studies for pinworms). For sample sizes of 100 power is generally low but higher for the ML estimation. For sample sizes of 600, the maximum likelihood analysis has superior power throughout and especially at the relevant effect sizes. Heatmaps on panels d–f depict the power across all analyzed sample and effect sizes for the three statistical tests

We suppose that technical and statistical differences between the studies seem more likely to explain the observed discrepancies. Most importantly, the more recent studies were examining 6‐ to 10‐fold larger sample sizes. We here performed a power analysis showing that such an increased sample size increases power substantially (Figure 3), irrespective of the statistical test employed in the study. Generally, low power can produce spurious results in sign, as effect sizes might be overestimated to an extent changing their direction. Type S (for sign) errors refers to the wrong identification of the sign of a comparison (Gelman & Tuerlinckx, 2000). Using few samples and noisy measurements in order to study small effects can easily lead to a result surprisingly likely to be in the wrong direction and to greatly overestimate an effect (Dumas‐Mallet et al., 2017; Gelman & Carlin, 2014).

Furthermore, the more recent studies within the HMHZ have used a hybrid index based on genetics (i.e., proportion of alleles from one mouse subspecies in a set of diagnostic markers, giving an estimate of the degree to which novel combinations of alleles are brought together in an individual compared to the pure subspecies; Baird et al., 2012) as a continuous scale for hybridization. This method for quantifying admixture can be shown to provide superior power (Figure 3), confirming that dichotomization of continuous variables, the practice of converting data sampled along a continuum into categories, can be harmful to data analysis (MacCallum et al., 2002). Moreover, both earlier studies used the categorical approach with different thresholds, the more stringent (Moulia et al., 1991) considering that a mouse presenting between 20% and 60% of Mmd alleles constitutes a hybrid, the more relaxed (Moulia et al., 1993) 2% to 97%. In our system, if there is an effect of hybridization on immune genes, hybrid resistance or susceptibility must be higher in the most introgressed mice compared to even slightly less introgressed ones (Baird et al., 2012). Dichotomization of hybrid index ignores this relationship. As the more current approach is a parametric statistical model, it is possible to consider further factors such as host sex (as employed in Baird et al., 2012 and Balard, Jarquín‐Díaz, Jost, Martincová, et al., 2020). This means one could also consider different effects of hybridization along environmental variables such as season or temperature gradients, for a spatial structure in the data or even for different parasite species in the same model.

Beyond the statistical issues we can ask how generalizable the results of the reviewed field are. Until very recently those studies focused on helminths. Extracellular macroparasites such as helminths trigger mainly a T‐helper 2 cell (Th2) immune response (Maizels et al., 2004; but also see Zhang et al., 2022). The effect of hybridization in terms of immune defenses of hybrid mice against parasites relative to mice from parental subspecies (higher, lower, or average) could depend on the type of immune response triggered. For this reason, we chose to focus our work on intracellular microparasites triggering a T‐helper type 1 (Th1)‐mediated response, *Eimeria* (Ehret et al., 2017). In Balard, Jarquín‐Díaz, Jost, Martincová, et al. (2020) we thus considered *Eimeria* spp. in addition to pinworms. We can argue that the latest study might offer a broader ground for generalization as different pathogens are assessed jointly.

As the latest refinement in the assessment of parasites we investigated parasite intensity in Balard, Jarquín‐Díaz, Jost, Martincová, et al. (2020). This means that we assess the extent of parasite infection only in infected animals (Bush et al., 1997). Absence of a parasite in a given host can be explained by several mechanisms: absence of exposure, complete host resistance, recovery, or death (Krämer et al., 2010). It would, for example, be conceptually possible that both subspecies, as well as their hybrids, differ in their ability to avoid parasitism. Laboratory‐raised female *Mus musculus domesticus* can discriminate males infected by *Eimeria vermiformis* from non‐infected males (Kavaliers & Colwell, 1995). This might hypothetically be used to avoid infection, even though avoidance of feces from parasitized individuals was not found when investigated in two wild rodents (namely white‐footed mice, *Peromyscus leucopus*, and deer mice, *Peromyscus maniculatus* (Walsh et al., 2013). A null parasite load could also result from ecological factors, for example, low parasite transmission due to a low density of hosts in a given sampling area. In natural systems, this would be particularly complicated to disentangle from intrinsic factors like parasite avoidance. When focusing on evidently parasite‐exposed animals and controlling simultaneously for the absence of differences in prevalence and mortality, it can be argued that we exclude most ecological factors that could contribute to parasite load differences. This approach prioritizes intrinsic host or parasite components and their interactions, and is therefore more targeted to the detection of effects of hybridization on resistance or susceptibility to parasites. In other words, this approach is more specific to the detection of host‐intrinsic effects than considering parasite abundance.

The pioneering study of Sage et al. (1986) raised a fascinating question regarding the possible role of parasites in the hybridization process. About this first work, however, Klein (1988) wrote that “the data are too preliminary to qualify for inclusion in a textbook.” He qualifies the conclusion of this study “a finding that still awaits confirmation on a truly representative sample.” We hold the opinion that the limitations of sampling techniques, the low sample size and statistical methods are the main reasons for the observed discrepancies in the follow‐up work. In our view fluctuations of hybrid effect over time and space are not likely, meaning that current evidence from field data suggests that hybrids in the HMHZ are more resistant to parasites than parental subspecies in general.

The different studies disagree on a second point, though: the role of parasites as a source of selective pressure. Indeed, to understand the possible impact of parasites on animals in the HMHZ, one must answer the question: does a change in parasite load necessarily imply a change in fitness?

## 4 STUDIES OF FITNESS EFFECTS OF PARASITES SHOULD INVESTIGATE RESISTANCE, HEALTH, AND TOLERANCE IN CONCERT

Most parasites reduce host fitness by impacting their health or—as the most extreme reduction of health—inducing mortality (Schmid‐Hempel, 2011; Figure 1). Other parasites have a more direct impact on reproduction, for example, by castrating their hosts (see Guttel and Ben‐Ami (2014) for an example in which this was investigated under the influence of hybridization), we note that castrating parasites are not known in house mice as the focus of this review. We thus only consider a fitness effect of parasite infection via two more direct components of fitness: (1) as a consequence of reduced health leading to reduced survival and (2) as a consequence of reduced reproduction, as infected individuals might experience anti‐parasitic mate choice (Ehman & Scott, 2002) or might have to forgo reproduction due to the allostatic load imposed on them (Korte et al., 2005). We here use “parasite health effect” in this broad sense to encompass all parasite effects on fitness by definition.

Hosts can defend themselves against parasitic infections in numerous ways. Resistance is the ability of a host to reduce its pathogen burden. It results from host defense against infection or proliferation (Kutzer & Armitage, 2016) and can imply behavioral or physiological mechanisms (Amoroso, 2021). When the immune response targeted at the parasite causes disease to the host (immunopathology), resistance can reduce host health and thus fitness too (Miller et al., 2005). To deal with both the direct damages created by parasite infection and immunopathology, a second category of defense mechanisms comes into play. Disease tolerance (not to be confused with immune tolerance which is the unresponsiveness of an immune system to a pathogen) is the ability of a host to reduce the damage induced by a certain parasite burden (Råberg et al., 2008). Tolerance has a direct, positive effect on health (morbidity and mortality tolerance) or more indirectly on fecundity (sterility tolerance) (Best et al., 2008). Contrary to resistance, tolerance can also increase parasite fitness, for example, by providing parasites with a longer living host niche (Kutzer & Armitage, 2016; Miller et al., 2005; Roy & Kirchner, 2000).

Unfortunately, only a few studies focusing on parasites as selective factors in hybridizing systems measure jointly resistance and tolerance in hybrids compared to parents. To our knowledge in only one example interpretable as tolerance investigation, in the freshwater snails genus *Melanopsis*, resistance against trematodes was found higher in hybrids than in parental taxa, and damaging parasite‐induced gigantism (a measure of tolerance) was absent in hybrids and present in all parental taxa (Guttel & Ben‐Ami, 2014).

Research into the evolutionary ecology of resistance and tolerance has classically been dominated by non‐mammalian models (Kutzer & Armitage, 2016) including plant–pathogen systems (Baucom & Roode, 2011). If the focus was long on resistance, disease tolerance is currently becoming a fertile research area, with publications arising on a variety of mammals, including our own work on *Eimeria* in mice (Balard, Jarquín‐Díaz, Jost, Martincová, et al., 2020), macroparasites in bank voles (Wanelik et al., 2018), PRRS virus in domestic pigs (Knap & Doeschl‐Wilson, 2020), or nematode infection in Soay sheep (Hayward et al., 2014), to cite a few. When taking a slightly more mechanistic perspective, tolerance and resistance on the host side are realized by the immune system and this is arguably best understood (and most relevant for humans) in mammals, especially in house mice as the predominant model in biomedical research (Yang et al., 2011). In addition to the impact of parasites on hybrid hosts this provides another reason to study resistance and tolerance jointly as an immunological phenomenon in house mice.

## 5 EXPERIMENTAL INFECTIONS OF HYBRID HOUSE MICE

Mice can be assessed easily in experiments and a number of studies report experimental infections of hybrids crosses between the two subspecies found in the HMHZ (Table 2). A first focus of these studies was obviously to test hybrid resistance, that is, susceptibility versus resistance based on parasite load differences, after the corresponding observations had been made in field studies. Regarding resistance of hybrids against infection these experimental infections can be regarded inconclusive:

**TABLE 2:** Laboratory studies using experimental infections to compare hybrids to parental subspecies of house mice

Reference	Parasite and its characteristics	Origin of mice	Number of mice	Hybrid definition	Response variable and statistical test	Result
Moulia et al. (1993)	Digestive helminths (low virulence)	Mmd: France Mmm: Georgia Hybrids: Denmark	156	**Categorical** Hybrid index based on 10 diagnostic markers Hybrid = HI between 2% and 97% of Mmd introgression	Individual parasite load Kruskal‐Wallis test & Noether's post‐hoc test between Mmd, Mmm & hybrid	Hybrid susceptibility
Moulia et al. (1995)	Digestive helminths (low virulence)	Mmd: France Mmm: Austria & Georgia Hybrids: crossing between the previous	290	**Categorical** Laboratory F1 crossing between Mmd and Mmm	Two load categories (wormy/not wormy) Fisher's exact test	Hybrid resistance
Derothe et al. (1999)	Blood protozoan (low virulence)	Mmd: Algeria, Morocco & Italy Mmm: Hungary & Poland Hybrids: Denmark & Bulgaria	261	**Categorical** Hybrid index based on 10 diagnostic markers Hybrid = HI between 2% and 89% of Mmd introgression	Individual parasite load Kruskal‐Wallis test & Noether's post‐hoc test between Mmd, Mmm & hybrid	No hybrid effect on resistance
Derothe et al. (2001)	Sarcoystis (using mice as intermediate hosts)		149			Hybrid susceptibility
Derothe et al. (2004)	Digestive helminths (low virulence)	Mmd: Algeria & Morocco Mmm: Hungary‐ Hybrids: crossings of the previous	805	**Categorical** Laboratory F1 to F4 crossings between Mmd and Mmm		Hybrid resistance

Note

Red: hybrid susceptibility; Green: hybrid resistance; Grey: no hybrid effect on resistance.

In total three laboratory studies followed the early field studies to use pinworms (Derothe et al., 2004; Moulia et al., 1993, 1995). While the earliest of these studies, with the smallest sample size, found increased pinworm load in hybrids (Moulia et al., 1993), the two following studies (Derothe et al., 2004; Moulia et al., 1995) found reduced parasite loads in hybrids. Derothe et al. (2004) showed that the reduction in parasite loads in hybrids extended to later recombinant F3 and F4 crosses. Two other studies used different parasites (potentially in an attempt to generalize the effect beyond pinworms, as we argue above for our own field observations). For *Sarcocystis muris* hybrid susceptibility was reported on the basis of a higher number of tissue cysts in hybrids (Derothe et al., 2001). This parasite likely occurs at very low prevalence in the wild (no reports are available, especially within the HMHZ) and uses the house mouse as one of several intermediate hosts. Similarly, *Trypanosoma musculi* (Derothe et al., 1999; found to have a similar load in hybrids than in parents) has a very low virulence and prevalence in the wild is not known, especially in the HMHZ.

These parasites are thus unlikely to co‐evolve with the house mouse or even to exert a sizable selective pressure on house mice in the wild. For this reason, it is unlikely to observe either (1) hybrid resistance by recombination of “parasite‐response alleles” or (2) hybrid breakdown by incompatibilities of such alleles. Altered response to a non‐specific parasite would thus be affected only by (3) generally altered physiology and responses to infection (e.g., general “hybrid vigor”). This means that only specific parasites are affected by all three possible mechanisms potentially underlying differences between hybrids and pure subspecies.

As an additional complication the mice used for experimental crosses to generate hybrids and parental subspecies controls were not derived from close to the HMHZ calling into question whether the experimental crosses would have the respective “parasite‐response alleles” even if those parasites were prevalent in the HMHZ.

The potential effects of parasitism on fitness of mice in the HMHZ have only been investigated based on resistance against parasites, that is, not incorporating more direct measures of health. As we outlined above in general terms, a combined assessment of resistance, health and tolerance is needed to estimate a fitness effect of parasites more clearly. The study of tolerance, however, needs robust assessment of the health effect of an infection (Råberg et al., 2008). As health status without a focal infection is unknown in the field it is very difficult to estimate the health effect of an infection under natural conditions. In the laboratory, however, health effects can be easily measured by, for example, comparing body condition before and during infection or the relative weight loss during infection. There are two reasons for experimental studies on hybrid mice restricting their analysis to resistance. Firstly, the attention to tolerance is relatively new in evolutionary ecology (as explained above), and secondly, the pathogens employed in previous studies are so lowly virulent that health effects might not be measurable (as e.g., weight loss or changes in body condition).

We conclude that an experimental focus on health as a fitness component requires suitable host–parasite models. The parasite should have a known, relatively high prevalence in the HMHZ, should have a sizable virulence, a narrow host spectrum and ideally a single host lifecycle. All of these factors make a parasite more likely to co‐evolve with the host, the host more likely to respond to selective pressure imposed by infections and the parasite more usable in the laboratory. An additional asset for such a model would be the availability of related parasites showing various levels of virulence in the same host. We recently investigated infections of house mice in natural settings and thus propose a natural parasite of house mice with moderate to relatively high virulence and still considerable prevalence in the HMHZ as such a suitable experimental model.

## 6 *EIMERIA* SPP. AS NEW MODEL PARASITES TO ASSESS RESISTANCE, HEALTH, AND TOLERANCE OF HYBRID MICE

In a recent study performed in the HMHZ, three *Eimeria* species were identified: *E*.* ferrisi*, *E*.* falciformis*, and *E*.* vermiformis* with prevalences of 16.7%, 4.2%, and 1.9%, respectively (Jarquín‐Díaz et al., 2019). The two most prevalent *Eimeria* species, *E*.* ferrisi* and *E*.* falciformis*, present in close ecological niches (*E*.* ferrisi* infects the cecum villar epithelial cells and *E*.* falciformis* the cecum crypt cells) (Ankrom et al., 1975; Jarquín‐Díaz et al., 2019). Importantly the two parasites show different virulence in laboratory infections. More precisely, the life cycle of *E*.* ferrisi* is shorter than that of *E*.* falciformis* (Ankrom et al., 1975; Balard, Jarquín‐Díaz, Jost, Mittné, et al., 2020). While both species provoke similar symptoms in laboratory mice, mainly diarrhea, lesion of the enteric epithelium, and weight loss (Al‐khlifeh et al., 2019; Balard, Jarquín‐Díaz, Jost, Mittné, et al., 2020; Ehret et al., 2017) we observed a higher virulence (induction of weight loss) for *E*.* falciformis* than for *E*.* ferrisi*. Higher weight loss and mortality induced by *E*.* falciformis* are likely correlated with a stronger immunopathology (Al‐khlifeh et al., 2019). Overall both species induce short infections associated with weight loss and can thus be considered relatively pathogenic compared to the most prevalent helminths (pinworms), which are present in chronic infections in the wild and do not induce weight loss in the laboratory. Given the high prevalence in the wild, compared to other virulent parasites the combination of high prevalence and virulence is likely to make *Eimeria* spp. a relevant source of selection pressure in the wild, which constitutes another reason to study this parasite in the HMHZ.

The genus *Eimeria* belongs to the phylum Apicomplexa, which contains only parasites. Their host range is extremely wide and includes birds, mammals, reptiles, amphibians, and fish (Chapman et al., 2013). They are described particularly well in domestic animals due to their economic importance, especially in poultry (Blake & Tomley, 2014), but can also be found in wild animals, where they are potentially problematic for conservation (Jeanes et al., 2013; Knowles et al., 2013; Matsubayashi et al., 2018). Each of the >1800 described *Eimeria* species is generally considered strictly host specific (Duszynski, 2011), but the recent use of multilocus genetic markers method in rodents showed that this host specificity could be less strict than previously thought (Jarquín‐Díaz et al., , 2020). The *Eimeria* life cycle presents both asexual (schizogony) and sexual (gametogony) phases, and takes place in a single host (Burrell et al., 2020). *Eimeria* oocysts, the infectious stage, are released in the environment via the feces and infect the next host by oral‐fecal contamination. The parasites infect epithelial digestive cells of their hosts, which leads to malabsorption of nutrients and weight loss.

*E*.* falciformis* (precisely, the isolate BayerHaberkorn1970 (Haberkorn, 1970)) is the most commonly used model for murine *Eimeria*. Host defense mechanisms against this parasite are well studied (Mesfin & Bellamy, 1979; Pogonka et al., 2010; Schmid et al., 2013) and its whole genome is sequenced and annotated (Heitlinger et al., 2014). T cells have been shown to play a major role in the defense against *E*.* falciformis* infection (Mesfin & Bellamy, 1979; Stiff & Vasilakos, 1990). Following infection, interferon γ (IFNγ) is upregulated (Schmid et al., 2013) and experimental infections showed higher weight loss and pathology but lower oocysts shedding in IFNγ‐deficient mice than in wild type (Stange et al., 2012). IFNγ alters the slope of the relationship between parasite burden and heath. This means that by definition IFNγ is a tolerance factor preventing increased immuno‐pathology resulting from the IL‐17 pathway when investigated in a reductionist knock‐out. Whether increased IFNγ expression would favor tolerance in natural populations is a different and unanswered question. Ehret et al. (2017) compared host and *E*.* falciformis* transcriptomes (dual transcriptomes) in immunocompetent and immunodeficient laboratory mice, and in naïve and challenged laboratory mice. They did not find differences in the gene expression profile of this parasite between differently immunocompetent hosts, and concluded that *E*.* falciformis* does not respond plastically to the host environment but rather present a genetically canalized (“hard wired”) program of infection.

**FIGURE 4:**
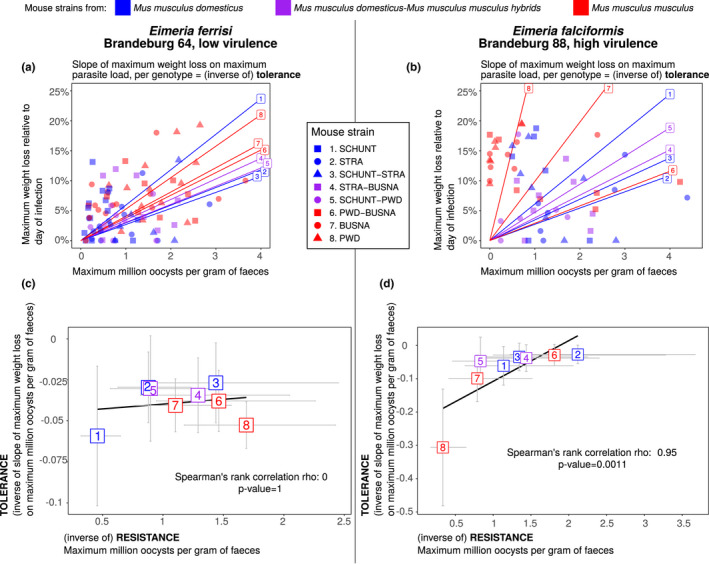
No evidence of coupling between resistance and tolerance in *Eimeria ferrisi* isolate Brandenburg 64, but support for this coupling for *Eimeria falciformis* isolate Brandenburg 88. (a, b) Tolerance between mouse groups estimated by the slope of the linear regression with null intercept modeling maximum relative weight loss as a response of maximum number of oocysts per gram of feces, a steep slope corresponding to a low tolerance. For *E*.* ferrisi* (A), the maximum number of oocysts per gram of feces differs between mouse groups, but tolerance is similar. For *E*.* falciformis* (b), both maximum number of oocysts per gram of feces and tolerance differ between mouse groups. (c, d) Correlation between maximum oocysts per gram of feces used as a proxy for (inverse of) resistance and tolerance. *E*.* ferrisi* does not shows a correlation (c), while we observe a strong positive correlation in *E*.* falciformis* (d) between maximum oocysts per gram of feces used as a proxy for inverse of resistance and tolerance (corresponding to a negative correlation between resistance and tolerance). Error bars represent 95% confidence intervals. Colors represent mouse subspecies (blue: *Mus musculus domesticus*, red: *Mus musculus musculus*, purple: Mmd‐Mmm), numbers in squares and point shapes represent different mouse strains. Figure adapted from Balard, Jarquín‐Díaz, Jost, Mittné, et al. (2020). This result is also schematized in Figure 5

**FIGURE 5:**
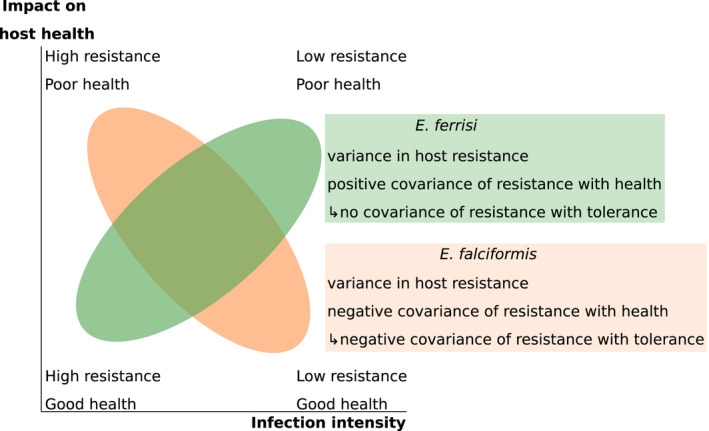
Different correlations between resistance and impact on health for infections with two different *Eimeria* species. Hosts infected with *E*.* ferrisi* present varying levels of resistance but similar tolerance, as the slopes of impact on health by infection intensity are not statistically different (see also Figure 4). Hosts infected with *E*.* falciformis* also present varying levels of resistance, but various tolerance slopes, and a negative resistance–tolerance correlation. This indicates a trade‐off between resistance and tolerance: more resistant hosts have a lower tolerance and less resistant hosts have a higher tolerance

In a recent infection experiment addressing resistance, health and tolerance together, the two different *Eimeria* species differed in their relationship between resistance and tolerance: In infection with *E*.* ferrisi* Mmd and Mmm strains, as well as F1 hybrids, showed different levels of resistance but very similar tolerance. In strong contrast, the different mouse strains showed a range of resistance levels for *E*.* falciformis*, but different levels of tolerance: Mmm mice tolerating the parasite badly, losing more weight for a given parasite load than Mmd and also succumbing to infections (Balard, Jarquín‐Díaz, Jost, Mittné, et al., 2020). In terms of plain parasite effects on health, this means that against the more pathogenic *E*.* falciformis* resistance and effects on health are negatively correlated, indicating immunopathology and a trade‐off in the reaction. For the less pathogenic *E*.* ferrisi* a rather positive correlation is found, indicating that resistance leads to better health (Figure 4; schematized in Figure 5). In this system, resistance is calculated as the inverse of maximum oocysts per gram of feces, this measure being tightly correlated to the sum of oocysts along the infection. Tolerance is calculated within each mouse strain as the slope of the linear regression with null intercept modeling maximum relative weight loss as a response of maximum oocysts per gram of feces, a steep slope corresponding to a low tolerance (Balard, Jarquín‐Díaz, Jost, Mittné, et al., 2020).

The likely increased resistance of hybrids against *Eimeria* spp. might result from recombination increasing the diversity of allele combinations involved in immune reactions responsible for resistance or, alternatively or additionally, from general “hybrid vigor” (Baird et al., 2012) having physiological effects which, for example, allow more resources to be available for parasite resistance. The tolerance mechanisms involved in response to infection, however, differ between the two host subspecies. This makes it necessary to consider that tolerance could differ in hybrids in response to different parasite species. Parasites could still play a role as selective factor advantaging (or penalizing) hybrids in the HMHZ and a combined analysis of resistance, health, and tolerance, will help to understand the underlying mechanism.

## 7 CONCLUSION AND PERSPECTIVE

We here voice the opinion that the evidence on hybrid resistance versus susceptibility to parasites in the natural environment of the European (HMHZ) is rather conclusive: hybrid mice are, in our opinion, likely to be more resistant to parasites than parental host subspecies, and contrasting results of previous studies might result from technical and statistical limitations.

We further argue that linking differential resistance directly to differential fitness in hybrids compared to parents is a dangerous shortcut. Differences in correlation between resistance and health effect of infection, that means tolerance, could distort the coupling between parasite load and effect on fitness. Recent work found differences between closely related *Eimeria* species in exactly this coupling of resistance and tolerance between two closely related parasites in laboratory infections. We conclude that it is necessary to measure resistance and tolerance jointly in experimental infections before drawing conclusions on the impact of parasitism on host health and extending these to host fitness.

In future, relative tolerance in hybrids compared to parental mice should be assessed in a controlled setting. We demonstrate and argue why we regard *Eimeria* spp. as a promising model parasite in this respect. Future work will allow to better understand the impact of parasites on hybrid host fitness. A parasite modulation of overall fitness might, while only modulating stronger effects of reproductive fitness (Figure 1), still be very relevant for the strength of introgression of mechanistically relevant genetic loci and for the overall effect of parasites on hybridization. Traditionally, research on pathogens in hybrid zones is focused on the evolution and ecology of the host and how this is impacted by the parasite. The effects of parasites on species barriers, for example, has been reviewed recently by Theodosopoulos et al. (2018), which led Baird and Goüy de Bellocq (2019) to criticize a naïve view of fitness differences between hybrids necessarily leading to changes in the extent of reproductive isolation. Turning the question around, to ask how parasites are impacted by HZ is an important new perspective (Goüy de Bellocq et al., 2018). This novel approach will also benefit from incorporating different aspects of the host immune defenses both mechanistically and conceptually in terms of resistance and tolerance.

## CONFLICT OF INTEREST

All the authors state that there is no conflict of interest

## AUTHOR CONTRIBUTIONS

**Alice Balard:** Conceptualization (equal); Data curation (equal); Formal analysis (equal); Investigation (equal); Software (equal); Writing – original draft (equal); Writing – review & editing (equal). **Emanuel Heitlinger:** Conceptualization (equal); Data curation (equal); Formal analysis (equal); Funding acquisition (lead); Investigation (equal); Methodology (equal); Project administration (lead); Resources (lead); Software (supporting); Supervision (lead); Visualization (equal); Writing – original draft (equal); Writing – review & editing (equal).

### OPEN RESEARCH BADGES

This article has been awarded Open Materials, Open Data Badges. All materials and data are publicly accessible via the Open Science Framework at https://github.com/derele/HMHZ_review.

## Data Availability

Data for this review is available in original research papers as cited. Code for the power analysis presented in Figure 3 is available at https://github.com/derele/HMHZ_review.
